# Portable Electronic Nose for Analyzing the Smell of Nasal Secretions in Calves: Toward Noninvasive Diagnosis of Infectious Bronchopneumonia

**DOI:** 10.3390/vetsci8050074

**Published:** 2021-04-27

**Authors:** Tatiana Kuchmenko, Anastasiia Shuba, Ruslan Umarkhanov, Anton Chernitskiy

**Affiliations:** 1 Faculty of Ecology and Chemical Technology, Voronezh State University of Engineering Technologies, 19 Revolution Avenue, 394000 Voronezh, Russia; Kuchmenko@vsuet.ru (T.K.); Shuba@vsuet.ru (A.S.); 2 Laboratory of Diseases of the Reproductive Organs, Breast and Young Farm Animals, All-Russian Veterinary Research Institute of Pathology, Pharmacology and Therapy, 114b Lomonosova Street, 394087 Voronezh, Russia; cherae@mail.ru

**Keywords:** calves, infectious bronchopneumonia, diagnosis, nasal secretions, electronic nose

## Abstract

The paper demonstrates a new approach to identify healthy calves (“healthy”) and naturally occurring infectious bronchopneumonia (“sick”) calves by analysis of the gaseous phase over nasal secretions using 16 piezoelectric sensors in two portable devices. Samples of nasal secretions were obtained from 50 red-motley Holstein calves aged 14–42 days. Calves were subjected to rectal temperature measurements, clinical score according to the Wisconsin respiratory scoring chart, thoracic auscultation, and radiography (Carestream DR, New York, USA). Of the 50 calves, we included samples from 40 (20 “healthy” and 20 “sick”) in the training sample. The remaining ten calves (five “healthy” and five “sick”) were included in the test sample. It was possible to divide calves into “healthy” and “sick” groups according to the output data of the sensor arrays (maximum sensor signals and calculated parameters A_i/j_) using the principal component linear discriminant analysis (PCA–LDA) with an accuracy of 100%. The adequacy of the PCA–LDA model was verified on a test sample. It was found that data of sensors with films of carbon nanotubes, zirconium nitrate, hydroxyapatite, methyl orange, bromocresol green, and Triton X-100 had the most significance for dividing samples into groups. The differences in the composition of the gaseous phase over the samples of nasal secretions for such a classification could be explained by the appearance or change in the concentrations of ketones, alcohols, organic carboxylic acids, aldehydes, amines, including cyclic amines or those with a branched hydrocarbon chain.

## 1. Introduction

Bovine respiratory disease (BRD) remains one of the leading causes of economic losses in dairy farming [1,2,3]. Among calves in the first month of life, BRD is recorded in 17.2–23.6% of cases [2,4]. The economic damage from BRD is derived not only from the direct cost of treating calves, as well as their culling and death [2,3,5], but also from the fact that, in the future, recovered heifers have a disordered reproductive function and a decrease in milk production [6,7,8]. BRD involves a group of heterogeneous pathologies (from rhinitis to severe pneumonia) [1,9,10], caused by a combination of genetic factors [11,12], physiological stressors (disturbances in feeding, microclimate parameters, transportation, regrouping, etc.) [13,14,15], and infectious agents [16,17], many of which may, however, be natural inhabitants of the respiratory tract in calves [16,18,19]. The complex nature of BRD and the absence of a universal “gold standard” decrease the probability to diagnose the BRD in a timely manner [10,20], as well as the development of optimal treatment and prevention regimens for BRD [4,21].

Several studies [10,22,23] demonstrated that a serious condition of calves with BRD, for example, infectious bronchopneumonia, can occur without obvious clinical signs. Since the clinical and laboratory diagnoses of infectious bronchopneumonia in calves have different accuracy, the development of alternative diagnostic methods is justified [24,25,26], primarily devices for the detection of BRD in calves directly on a farm.

One of the promising areas in the diagnosis of BRD in calves is metabolomics [24,27,28]. Metabolomics studies the changes in the concentrations of specific metabolites in tissues and biological fluids. The metabolite profiles help understand the disease’s pathogenesis, and they can be used as biomarkers for diagnosis [29,30,31,32,33]. The most perspective direction in BRD diagnosis using metabolomics involves noninvasive methods when researching the metabolic profiles of exhaled breath gas [34], exhaled breath condensate [35], and nasal secretions [28,36]. One of such diagnostic methods is the analysis of the composition of the gaseous phases of biological samples (exhaled breath condensate, nasal secretions) using an array of piezoelectric sensors with the methodology of an “electronic nose” (e-nose) [36,37]. Traditionally, the determination of BRD volatile markers in the gas phase over biological samples using an e-nose is carried out on the basis of sensor signals using pattern recognition algorithms or multivariate regression. The whole set of substances is evaluated as an integral indicator of the biological sample condition [38,39,40,41]. An alternative approach to processing the sensor array data in the analysis of biological samples is also possible, based on calculating the sorption efficiency parameters A_i/j_ [37,42,43], which mainly reflect the qualitative composition of the gas mixture and can be further processed by various mathematical algorithms.

In this study, we propose a technique to identify healthy calves (“healthy”) and naturally occurring infectious bronchopneumonia (“sick”) calves via analysis of the gaseous phase over nasal secretions using portable e-noses with piezoelectric sensors. Our working hypothesis is based on the conception that gas phases over nasal secretions have a specific signature of volatile organic compounds (VOCs) for calves diagnosed as “healthy” and “sick”.

## 2. Materials and Methods

### 2.1. Ethics Statement

The Ethics Committee of the Voronezh State University of Engineering Technologies approved all procedures for clinical examination of animals and obtaining samples for analysis used in this work (Minutes No. 2 dated 25 February 2021). The care and use of animals complied with Russian animal welfare laws, guidelines, and policies; the study did not affect normal animal physiology.

### 2.2. Animal Materials and Study Design

The research was performed during winter when the cattle were kept in stalls on a farm impacted by BRD (*Mycoplasma bovis* and bovine adenovirus 3). The objects of the study were 50 red-motley Holstein calves aged 14–42 days: 25 individuals with naturally occurring infectious bronchopneumonia (“sick”) and 25 clinically healthy animals (“healthy”). All calves we divided into two samples. The training sample included 40 calves (20 “healthy” and 20 “sick”, Table 1). The remaining 10 calves (five “healthy” and five “sick”) were included in the test sample (presented in Table 2).

All animals were examined according to the clinical scoring system developed by veterinarians at the University of Wisconsin at Madison (WI clinical score) [44] (measurement of rectal temperature, assessment of the presence of cough, nasal discharge, ocular discharge, head, and ear position). Lung lesions were detected using thoracic auscultation (Littmann^®^ Master Classic II Veterinary Stethoscope, 3M, Saint Paul, MN, USA) and radiography (Carestream DR, Carestream, New York, USA). The trachea was palpated [44,45], and a 30 s expiratory apnea was performed to induce cough in calves [22,46].

**Figure 1:**
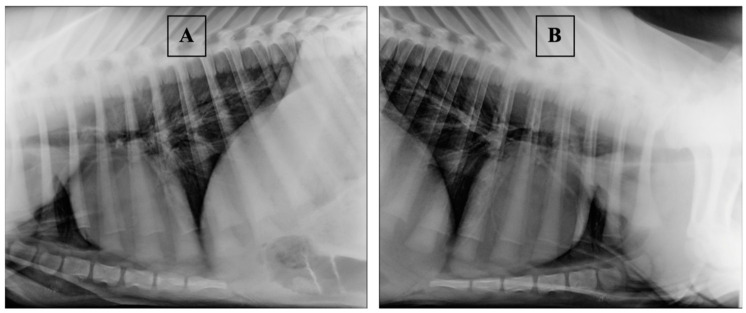
Typical radiographic images of “healthy” calves: (**A**) right lateral position; (**B**) left lateral position.

**Figure 2:**
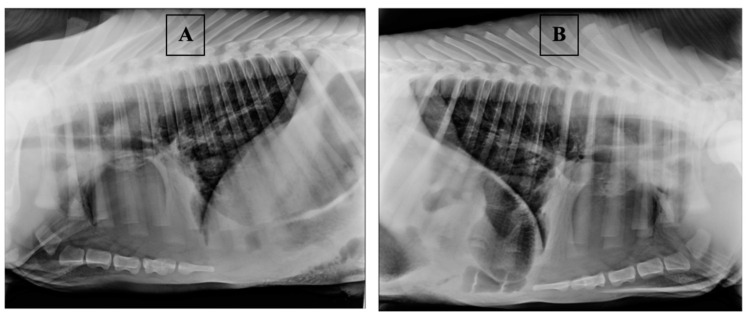
Typical radiographic images of “sick” calves: (**A**) right lateral position; (**B**) left lateral position.

Thoracic radiography in calves was carried out by fixing them in the supine position on the right and left sides, with the forelimbs extended cranially [47,48]; the exposure factors and source-to-image-receptor distance were set to 70 kV, 5.0 mAs, and 100 cm, respectively. Typical radiographic images of “healthy” calves are shown in Figure 1. In “healthy” animals, on radiographic images, the pulmonary fields’ airiness remained on the whole surface of the chest, the alveoli were filled up with air, the bronchi had good airiness, and the bronchial pattern was clearly visible in all pulmonary fields. Typical radiographic images of “sick” calves are shown in Figure 2. In “sick” calves, the cranial and (or) middle pulmonary fields were contracted, while the bronchi remained airless. In some cases, a slight focal contraction was observed along the bronchi in the caudal pulmonary field.

Bronchopneumonia was diagnosed in calves with a clinical score according to the Wisconsin respiratory scoring chart of 4 or more, spontaneous or induced cough, abnormal thoracic auscultation (crackles, wheezes, or absence of sound), radiographic signs of lung lesions [49,50]. As for “healthy” animals, the WI clinical score was 3 or less, whereas radiographic and clinical (spontaneous and induced cough, dyspnea, abnormal auscultation) signs of lung lesions were absent.

### 2.3. Collection of Samples

Samples of nasal secretions for analysis were taken from calves immediately after the completion of their clinical study. Two samples were obtained from each of 50 animals; a total of 100 samples were collected. A sampling of each nasal secretions was within 4–5 s consecutively from the left and right nasal passages using sterile cotton swabs, which are usually used for bacteriological research. The swabs (-) were used with the following properties: without medium, length of 150 mm, rod material—plastic, tip—cotton, flexible, sterile, and individually wrapped. Manufacturer LAB-Medica, Russia.

### 2.4. Volatile Organic Compounds Analyses

#### 2.4.1. Device and Sensor Array Characteristics

**Figure 3:**
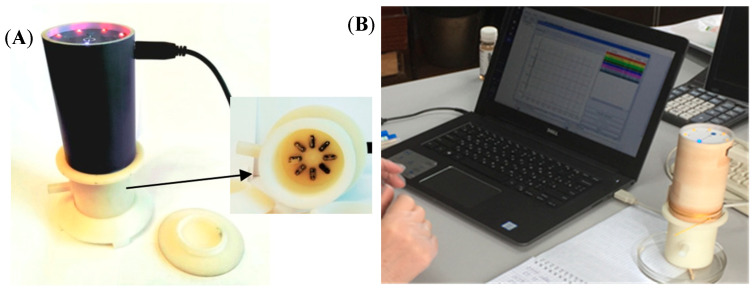
General view of analyzer “Diagnost-Bio-8” (**A**); during measurement of nasal secretion sample (**B**).

Analysis of gaseous phase over nasal secretions samples was carried out on the odor analyzer “Diagnost-Bio-8” (Ltd. «Sensino», Kursk, Russia, Figure 3). We used two devices with two sensor arrays. Each sensor array consists of eight piezoelectric quartz resonators of BAW-type with 10.0 MHz basic oscillation frequency. The electrodes of resonators were modified by various solid-state nanostructured sorbents and polymeric films. The modifiers for sensitive coatings of sensors are listed in Table 3.

These sorbents were chosen among 50 phases due to them having hypersensitivity to various classes of highly volatile organic compounds (alcohols, aldehydes, acids, ketones, amines, and arenes) [36,37,51,52,53,54,55,56,57], including volatile biomarkers of respiratory pathologies.

The sensor technique, including hydroxyapatite synthesis, and technical characteristics are specified in [55,58].

Baselines of sensors in arrays were stable (±1 Hz) during the active measurement time (80 s).

#### 2.4.2. Output Data of Sensor Arrays

**Figure 4:**
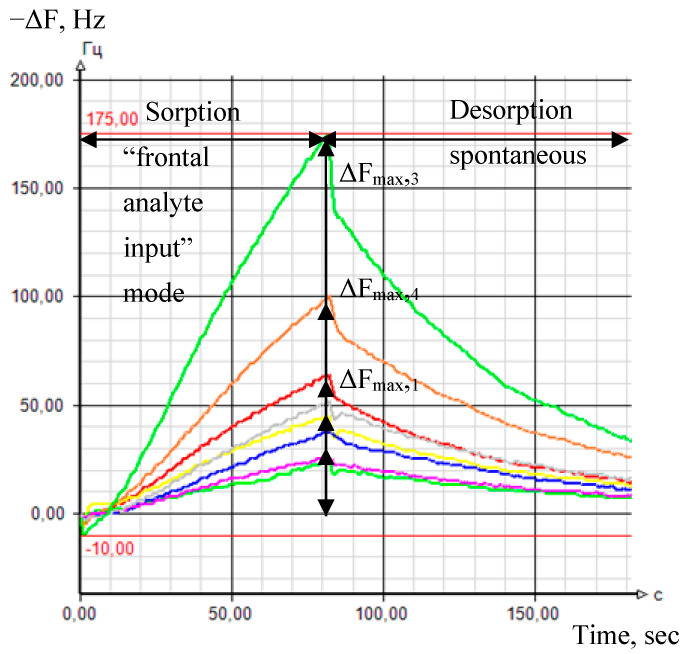
Chrono-frequency-grams of sensors from the first set at the measurement of nasal secretion sample.

**Figure 5:**
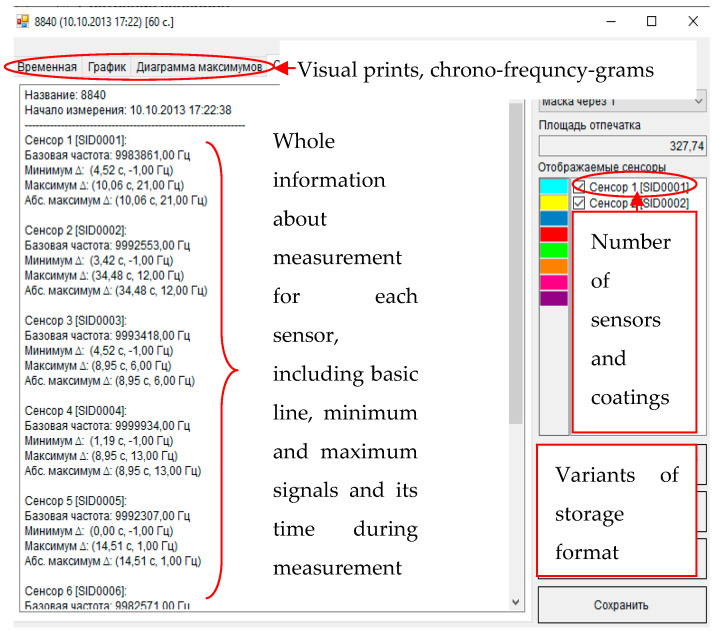
The dialog window of measurement in the program.

The sensor array’s primary analytical information is chrono-frequency-grams—output curves of piezoelectric quartz sensors within the total time of measurement—depending of alterations in the vibration frequency (−ΔF, Hz) of each sensor with time (Figure 4). The chrono-frequency-grams were recorded and stored in the special software developed by our group previously [59]. The device’s software is written in C# (C sharp); it uses the free regulatory database management system MySQL. The dialog window of the program after the measurement is presented in Figure 5. According to it, the program finds out the maximum sensor signals (ΔF_max,i_, Hz) during the sorption of the gas phase of biological samples (the first 80 s of measurement, Figure 4 and Figure 5).

The obtained maximum sensor signals were used to calculate the parameters of the sorption efficiency A_i/j_ [60] for each set of sensors as follows:

A_i/j_ = ΔF_max,i_/ΔF_max,j_,

where i and j are the number of sensors in the array.

The total number of parameters A_i/j_ for the two arrays was 56.

#### 2.4.3. Measurement Mode

Two samples of nasal secretions were obtained from each of the 50 calves. Nasal swabs were scrutinized in the “frontal analyte input” mode (spontaneous evaporation of highly volatile compounds from a sample in pre-sensory space of the detection cell) at 20 ± 1 °C. Within 20–30 min of selection, cotton swabs with nasal secretions were extracted from sterile tubes and placed on the glass Petri plate. Hereafter, the detection cell of “Diagnost-Bio-8” was used to tightly cover the plate with the sample (Figure 3B), and the measurement process was initiated. The time between the moment we removed the cotton swab from the tube and measurement itself was 10 s and was strictly controlled. The measurement mode was combined: sensors in the detection cell were kept above the sample during the first 80 s; at the 81st second, the device with an open detection cell was placed on a unique stand for spontaneous desorption of volatile compounds from the sensor coverings (Figure 3A). The total measurement time of one sample was 200 s; by this time, the vibration frequencies of the sensors returned to their baselines. After measurement with the first set of sensors, the sample was immediately measured using another device with a second set of sensors in the same measurement mode. Thus, all 100 samples were measured twice, once on each sensor array. Previously, using a similar measurement mode, we studied on all 16 sensors (in two sets) the sorption/desorption of vapors of distilled water [61] and 21 volatile compounds (ketones, arenes, aldehydes, amines) in concentrations from 1 ppmv up to 10 ppmv, with the estimation of parameters A_i/j_ for these substances (Table 4). These compounds are markers of respiratory pathologies established by a different research group [62,63,64,65].

### 2.5. Algorithm of Classification

The calculated parameters of the sorption efficiency A_i/j_ and the maximum signals of the 16 sensors for all samples were used to construct the initial data matrix of sensor arrays. The output data of the sensor arrays for each day of the study were autoscaled by the mean and standard deviation to reduce the influence of the sensor time drift. Linear discriminant analysis (LDA) with a significance level of 0.05 was selected as a method for classifying samples into diagnostic groups “healthy” and “sick”. Since all the variables in the LDA method should be independent, and the parameters A_i/j_ correlate a priori with each other and with the sensor signals, the entire initial data matrix before the LDA was processed by principal component analysis (PCA), and seven principal components were selected to construct the discriminatory functions with an explained variance of 80%. The data matrix was processed using the module for Microsoft Excel and Unscrambler X 10.0.1 (CamoSoftware AS, Oslo, Norway) with the possibility of sequential processing by principal component and linear discriminant analysis (PCA–LDA).

The output data of sensor arrays from the analysis of the gas phases over nasal secretions samples for 40 calves (*n* = 20 for “healthy” and “sick”, averaged values of two samples from each animal) from the training sample were used to constructed the PCA–LDA model to classify samples into diagnostic groups “healthy” and “sick”. The overall matrix dimension for constructing model was 40 × 72 (it is presented in the Supplementary Materials), while that after processing by PCA was 40 × 7. We used output data of sensor arrays for nasal swabs from 10 calves (averaged values of 2 samples from each animal) of the test sample to check the adequacy of the obtained mathematical model. The initial matrix dimension for the test sample was 10 × 72, while that after implementation of PCA was 10 × 7.

## 3. Results

In the training sample, the clinical WI score for “healthy” calves was 1.6 ± 0.6 (range from 0–2 points), while “sick” animals had a score of 6.3 ± 1.7 (range from 4–10 points). Among “sick” calves, 85% had a WI clinical score of 5 or more, while 15% had a WI clinical score of 4. All “sick” calves had abnormal thoracic auscultation (crackles, wheezes, or absence of sound) and radiographic signs of lung lesions. The rectal temperature in “healthy” calves was 38.9 ± 0.4 (38.1–39.4) °C, whereas that in “sick” animals was 39.2 ± 0.7 (38.3–41.7) °C. Hyperthermia (over 39.5 °C) was observed in 20% of “sick” calves. Spontaneous and induced cough was absent in the “healthy” calves. As for “sick” calves, in 95% of cases, a cough was observed, induced by the trachea’s palpation, in 85% of cases, by 30 s apnea. We noted spontaneous cough in 80% of cases.

**Figure 6:**
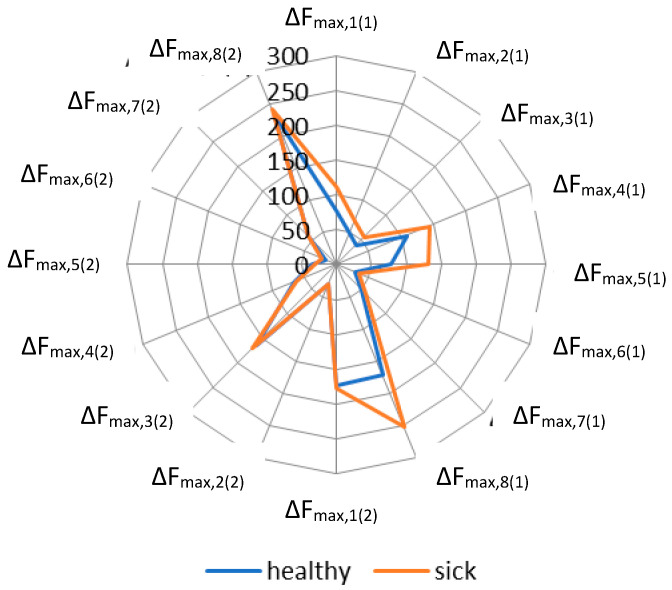
The “visual prints” of averaged signals of sensors for the diagnostic groups of calves.

To assess the possibility of separating samples of nasal secretions with or without BRD using a portable electronic nose, “visual prints” of diagnostic groups were constructed on the basis of averaged sensor signals (Figure 6).

The coefficient of variation of sensor signals was calculated to compare the variance of signal values for nasal secretion samples from different diagnostic groups (Table 5).

**Figure 7:**
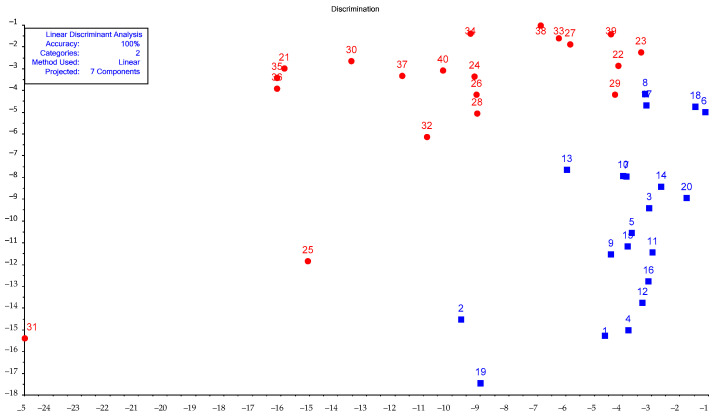
PCA–LDA plot to discriminate “healthy” (blue squares) and “sick” (red circles).

A mathematical model was obtained on the basis of the results of processing the output data of sensor arrays using PCA–LDA (Figure 7, also in the Supplementary Materials).

**Figure 8:**
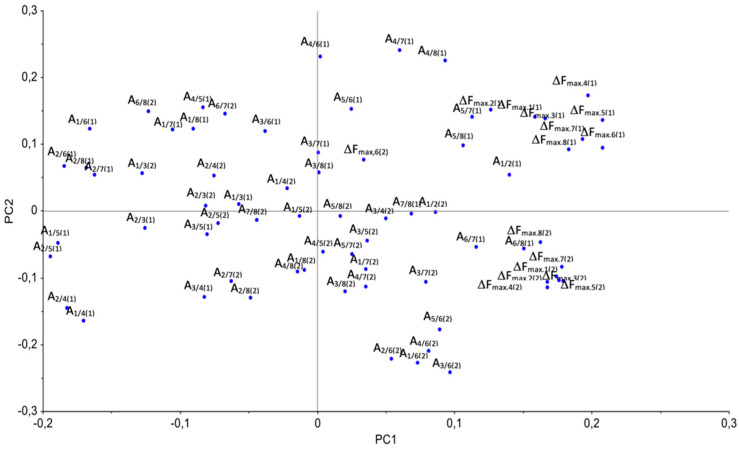
Loading plot for PC1–PC2 of the obtained PCA–LDA model.

For a training set of 40 samples, the calculated classification accuracy of the PCA–LDA model was 100%. The initial variables’ most significant contribution to the classification model was assessed by the loading values. Figure 8 shows the loading plot for the first two principal components. Table 6 presents the loading values for the remaining components.The loading values for seven principal components are presented in the Supplementary Materials.

We checked the robustness of the obtained model using sensor data for 10 calves from the test sample. As a result (Table 7), five samples were classified as “sick”, and five samples were classified as “healthy”, which entirely coincides with the results of clinical studies of these calves.

Thus, in the test sample, the WI clinical score of “healthy” calves was 2.4 ± 0.9 (range from 1–3 points), and “sick” animals had a score of 4.2 ± 0.4 (range from 4–5 points). Only 20% of “sick” calves had a WI clinical score≥5, whereas 80% had a WI clinical score of 4, and 100% of individuals had induced or spontaneous cough, abnormal thoracic auscultation, and radiographic signs of lung lesions. The rectal temperature in “healthy” calves was 38.9 ± 0.3 (38.6–39.4) °C, while that in “sick” calves was 38.5 ± 0.2 (38.3–38.8) °C.

## 4. Discussion

The choice of variables for modeling is an important step, on which the quality of the resulting model directly depends. In contrast to the standardly used maximum sensor signals, the calculated parameters of the sorption efficiency A_i/j_ are also used as variables in this work. The parameters A_i/j_, as shown earlier [60] are a measure of the relative sensitivity of two piezosensors to sorbate, and they depend, first of all, on the nature of the sorption system, thus mainly characterizing the qualitative composition of the gas mixture. It is shown that the parameters A_i/j_ can be used as identification parameters subject to certain conditions and assumptions. Previous studies [42,43,60,66] demonstrated that the fulfillment of these conditions is ensured when measuring the gas phases of biological samples using arrays of sensors, including those with the selected types of coatings.

The main assumptions and conditions included strict adherence to the methods of forming coatings on the electrodes of piezoelectric resonators, ensuring the linearity of the sensors’ response (constancy of the sensitivity of micro-weighing) in the selected range of concentrations of the detected substance. Earlier, it was found [60] that the variation of sensor signals is no more than 6.5%, which is acceptable when used to calculate criteria for the identification of substances [67]. The content of VOCs in the gas phase above biological samples, as a rule, does not exceed 1–10 ppm [62,64,65], which ensures the constancy of the sensitivity of micro-weighing. Consequently, the values of the parameters A_i/j_ do not depend on the concentration and are constant; thus, they can be used to identify substances. When identifying a substance using one parameter A_i/j_, it is necessary that the identification value of the parameter has a minimum or maximum value for a specific substance or a group of substances (homologs), and the difference between the closest values of this parameter A_i/j_ for different substances must be greater than 3 σ. When the coincidence of several parameters identifies a substance, it is possible to use parameters for several classes of substances. In that case, a substance or group is identified when at least two parameters for a given class of substances coincide. A different value of the coincidence criterion *d* (the confidence interval of the parameter values; when the parameter values calculated for the sample coincide with the tabular values within these boundaries, the substance is identified) is allowed with the homogeneity of the dispersion due to the different sorption affinity of sorbents for analytes, which determines the magnitude of the dispersion in the sorbate–sorbent systems. The calculated values of the parameters A_i/j_ and criterion *d* are determined for the given experimental conditions, taking into account the nature of volatile substances, the composition of the sample, and the mode of supplying the gas phase to the detection cell (Table 4). A more detailed list of identification parameters was presented in [66]. The detection limits, calculated with the implementation of the efficiency curves, for the substances are in the range of 0.8–40 ppm [60,66]. If the content of substances in the gas phase is above the indicated detection limits, the reliability is more than 99%.

The presence and quantitative content of pathogenic microorganisms, viruses, and inflammation products in the sample considerably change the composition of the gases excreted from the biosample [68,69,70,71]. Consequently, the inclusion of parameters A_i/j_ in the initial data matrix allows taking into account the presence or increase in the concentration of individual substances in the gas phase more comprehensively when constructing the classification model.

The principal component analysis preliminarily processed the sensor array output data to reduce the variables’ dimension and orthogonalization. For further construction of discriminatory functions, seven components with an explained variance of 80% were selected since we believe that the remaining 20% characterize noise when using an open detection cell with an array of sensors.

The plot of the obtained LDA model (Figure 7) shows that nasal secretion samples from “sick” calves had more variation within the group than samples from “healthy” calves, which reflects a greater variety of gaseous phase composition over nasal swabs in the case of BRD. This assumption was confirmed by the “visual prints” of averaged signals for the diagnostic group (Figure 6).

Figure 6 demonstrates that the averaged signals of the first set of sensors had large values for the “sick” group, especially for sensors with nanostructured coatings (sensors No. 1, 4, 5, and 8). The coefficient of variation of sensor signals for samples of calves from the “healthy” group was less than that in the “sick” group (up to the 42% and 60%, respectively; Table 5). Moreover, the difference in the variation coefficients of signals sensor with nanostructured coatings for diagnostic groups was bigger than for sensors with polymeric modifiers (37% and 17%, respectively).

The differences in average values of sensor signals with polymeric coatings are not significant, but slight shifts in signals of these sensors could be significant for classification model using chemometrics methods.

The significance of the model’s variables decreased in the order of increasing principal components because the percentage of explained variance for each subsequent principal component decreased. The loading plot (Figure 8) shows that the initial signals made the most considerable contribution to the model of the sensors ΔF_max,i_, and the signals from the first set of sensors were more significant than from the second set. Therefore, taking into account the loadings for the seven principal components of the PCA–LDA model (shown in Table 6, Figure 8), the most significant for classification were the signals of sensors with modifiers of carboxylated carbon nanotubes, zirconium nitrate, hydroxyapatite, methyl orange, bromocresol green, Triton X-100, and polyethylene glycol and its ethers, which are highly sensitive to vapors of nitrogen- and oxygen-containing compounds according to our previous investigation of sorption features of VOCs on these sorbents [37,51,52,53,54,55,56,57].

Among the parameters A_i/j_, the most significant to the classification model for the first two principal components were A_1/4(1)_, A_1/5(1)_, A_2/4(1)_, A_2/5(1)_, A_2/6(1)_, A_4/6(1)_, A_4/7(1)_, A_4/8(1)_, A_1/6(2)_, A_2/6(2)_, A_3/6(2)_, and A_4/6(2)_ (Figure 8). Consequently, the sensors with films of carboxylated and multiwalled carbon nanotubes, hydroxyapatite, and zirconium nitrate were most important for the classification model. According to the parameters A_1/4(1)_, A_2/6(1)_, and A_4/6(1)_ in the gaseous phase of nasal mucus from “sick” calves, carboxylic acids C_2_–C_4_ and ethanol were identified. According to the parameters A_1/5(1)_, A_2/6(1)_, and A_4/8(1),_ ketones, alcohols, and benzaldehydes were identified in more than 50% of samples from the “sick” group. The parameters A_1/3(2)_, A_1/5(2)_, A_2/3(2)_, A_3/5(2)_, A_5/7(2)_, and A_5/8(2)_ were also significant for the model according to values of loadings for the third principal component (Table 6). This means that signals of sensors with bromocresol blue (sensor 5) and methyl orange (sensor 3) are also significant for discriminating “healthy” and “sick” groups. In previous studies [52,53,54,55], we found that these coatings exhibit the most remarkable mass sensitivity to cyclic amines, aromatic amines, and carboxylic acids, respectively. Hence, when classifying samples into the “healthy” and “sick” groups, the appearance or change in the concentrations of ketones, alcohols, aldehydes, organic carboxylic acids, and amines, including cyclic amines or those with a branched hydrocarbon chain, in the gas phases over nasal swabs could be significant. The classification model’s high accuracy was confirmed by dividing the test set from 10 calves into “healthy” and “sick”. The sensitivity and specificity of the proposed classification model were 100% with a confidence level of 0.05. To clarify the composition of the gas phase over samples of nasal mucus from calves from different diagnostic groups and to assess the proposed approach’s sensitivity and specificity more accurately, we will conduct additional studies on a larger sample of animals.

## 5. Conclusions

Thus, the principal possibility of diagnosing infectious bronchopneumonia in calves on the basis of the smell of nasal secretions samples using 16 chemical sensors was demonstrated. The PCA–LDA model obtained from the output data of sensor arrays allowed the high-accuracy classification of samples into the “sick” and “healthy” groups, and it can be used for on-farm diagnosis of infectious bronchopneumonia in calves. The differences in the gaseous phase’s composition over the samples of nasal secretions for such a classification could be explained by the appearance or change in the concentrations of ketones, alcohols, organic carboxylic acids, aldehydes, and amines, including cyclic amines or those with a branched hydrocarbon chain. It is possible to simplify the technique for measuring nasal secretions samples by using one device and reducing the total number of sensors to eight by choosing the most informative ones for the PCA–LDA model. We will check this hypothesis in future research.

## Figures and Tables

**Table 1:** Training sample of calves.

Sample	Rectal Temperature, °C	Cough Score	Wisconsin Respiratory Scoring Chart (WI Clinical Score)	Group of Calves
1	38.1	0	0	Healthy
2	38.7	0	1	Healthy
3	38.6	0	1	Healthy
4	38.7	0	1	Healthy
5	38.7	0	1	Healthy
6	38.8	0	1	Healthy
7	38.8	0	1	Healthy
8	38.7	0	1	Healthy
9	38.3	0	2	Healthy
10	38.9	0	2	Healthy
11	39.2	0	2	Healthy
12	38.8	0	2	Healthy
13	39.3	0	2	Healthy
14	39.4	0	2	Healthy
15	39.0	0	2	Healthy
16	39.3	0	2	Healthy
17	39.2	0	2	Healthy
18	38.3	0	2	Healthy
19	39.1	0	2	Healthy
20	39.4	0	2	Healthy
21	39.6	3	6	Sick
22	38.6	2	4	Sick
23	38.3	3	4	Sick
24	38.8	3	4	Sick
25	38.9	3	5	Sick
26	39.4	1	4	Sick
27	39.3	3	5	Sick
28	39.4	3	5	Sick
29	38.4	3	6	Sick
30	38.9	3	7	Sick
31	38.8	3	7	Sick
32	39.3	3	7	Sick
33	39.3	3	7	Sick
34	38.9	3	7	Sick
35	39.0	3	7	Sick
36	39.8	3	7	Sick
37	39.4	3	8	Sick
38	38.4	3	8	Sick
39	40.0	3	8	Sick
40	41.7	3	10	Sick

**Table 2:** A test sample of calves.

Sample	Rectal Temperature, °C	Cough Score	WI Clinical Score	Group of Calves
41	38.8	0	1	Healthy
42	38.9	0	2	Healthy
43	39.4	0	3	Healthy
44	38.6	0	3	Healthy
45	39.0	0	3	Healthy
46	38.8	3	4	Sick
47	38.3	3	4	Sick
48	38.5	2	4	Sick
49	38.6	3	4	Sick
50	38.5	3	5	Sick

**Table 3:** The sorbents for sensitive coating of sensors.

Number of a Sensor in an Array	Sorbent	Origin
The first set		
1, 8	Carboxylated carbon nanotubes of different masses (1–5 μg)	Institute for Extra Pure Materials of the Russian Academy of Sciences, Russia, Moscow region, Chernogolovka
2, 7	Zirconium nitrate of different masses (1–5 μg)	Reachem, Moscow Russia, (puriss.)
3	Dicyclohexane-18-crown-6	Alfa Aesar, Ward Hill, USA, p.a.
4, 5	Hydroxyapatite of different masses (1–5 μg)	Own technique of synthesis
6	Polyethylene glycol succinate	Reachem, Moscow Russia, (puriss.)
The second set		
1	Polyethylene glycol 2000	Alfa Aesar, Ward Hill, USA, p.a.
2	Dicyclohexano-18-crown-6	Alfa Aesar, Ward Hill, USA, p.a.
3	Methyl orange	Reachem, Moscow Russia, (puriss.)
4	Triton X-100	Alfa Aesar, Ward Hill, USA, p.a.
5	Bromocresol blue	Reachem, Moscow Russia, (puriss.)
6	Multiwalled carbon nanotubes	Institute for Extra Pure Materials of the Russian Academy of Sciences, Russia, Moscow region, Chernogolovka
7	Polyethylene glycol sebacinate	Reachem, Moscow Russia, (puriss.)
8	Tween-80	Reachem, Moscow Russia, (puriss.)

**Table 4:** Parameters A_i/j_ for pure volatile substances.

Parameter	Values for Identification A_i/j_ ± d *	Identified Substances
A_1/4(1)_	0.95 ± 0.15	Carboxylic acids C_2_–C_4_
A_1/5(1)_	0.85 ± 0.04	Triethylamine, cyclopentylamine
	1.50 ± 0.50	Methylbenzaldehyde, benzaldehyde, formic acid
A_2/4(1)_	1.00 ± 0.10	Ethyl acetate, methylpropanone, acetone
	1.80 ± 0.40	Aliphatic, cyclic amines of normal and isomeric structure
A_2/5(1)_	1.65 ± 0.35	4-methylbenzaldehyde, benzaldehyde, acetone, formic acid
	3.5 ± 1.0	Cyclohexanone, m-methylcyclohexanone, cyclopentanone, 2-methylhexanone, acetaldehyde, C_2_–C_5_ alcohols of normal and isomeric structure
A_2/6(1)_	2.75 ± 0.75	Ketones, alcohols, carboxylic acids C_2_–C_4_
	1.50 ± 0.20	Methylbenzaldehyde, benzaldehyde, water, 2-thiophenecarbaldehyde
A_4/6(1)_	5.0 ± 0.2	Ethanol
A_4/8(1)_	0.25 ± 0.11	Ketones, alcohols, benzaldehyde, methylbenzaldehyde, ethyl acetate, acetaldehyde
	0.75 ± 0.15	Water, methylamine

* *d*—criterion of coincidence.

**Table 5:** The coefficients of variance of sensors signals for nasal secretion’s samples of calves from diagnostic groups.

Coatings	Healthy	Sick	Coatings	Healthy	Sick
Carboxylated carbon nanotubes 1 *	15	45	Polyethylene glycol 2000	25	27
Zirconium nitrate 1	16	45	Dicyclohexano-18-crown-6	22	27
Dicyclohexane-18-crown-6	18	59	Methyl orange	32	36
Hydroxyapatite 1	23	47	Triton X-100	24	30
Hydroxyapatite 2	23	61	Bromocresol blue	23	45
Polyethylene glycol succinate	19	31	Multiwalled carbon nanotubes	42	47
Zirconium nitrate 2	17	21	Polyethylene glycol sebacinate	24	30
Carboxylated carbon nanotubes 2	19	58	Tween 80	30	25

* 1 and 2 are designated coatings with different masses.

**Table 6:** Loading values for sensor output data in PCA–LDA model.

PC	ΔF_max,1(1)_	ΔF_max,2(1)_	ΔF_max,3(1)_	ΔF_max,4(1)_	ΔF_max,5(1)_	ΔF_max,6(1)_	ΔF_max,7(1)_	ΔF_max,8(1)_
3	0.059	0.061	0.054	0.071	0.111	0.114	0.110	0.040
4	0.186	0.179	0.102	0.090	0.141	0.124	0.140	0.144
5	−0.104	−0.172	−0.096	−0.066	−0.061	−0.127	−0.120	−0.112
6	0.107	0.052	0.122	0.084	0.066	0.144	0.114	0.112
7	0.045	0.043	−0.095	0.032	0.031	0.035	0.056	0.110
PC	ΔF_max,1(2)_	ΔF_max,2(2)_	ΔF_max,3(2)_	ΔF_max,4(2)_	ΔF_max,5(2)_	ΔF_max,6(2)_	ΔF_max,7(2)_	ΔF_max,8(2)_
3	−0.169	−0.145	−0.186	−0.148	−0.112	−0.215	−0.163	−0.206
4	0.048	0.045	0.049	−0.015	−0.015	0.102	0.025	0.012
5	−0.073	−0.078	−0.057	−0.074	−0.141	−0.151	−0.135	−0.076
6	−0.140	−0.169	−0.119	−0.144	−0.197	−0.151	−0.186	−0.168
7	0.006	−0.004	−0.004	0.008	−0.016	0.008	0.026	0.094
PC	A_1/2(1)_	A_1/3(1)_	A_1/4(1)_	A_1/5(1)_	A_1/6(1)_	A_1/7(1)_	A_1/8(1)_	A_2/3(1)_
3	−0.009	0.057	−0.051	−0.123	−0.077	−0.125	−0.027	0.036
4	0.036	0.115	0.211	0.087	0.124	0.116	0.257	0.060
5	0.139	0.031	−0.137	−0.139	0.106	0.090	0.087	−0.069
6	0.201	−0.062	0.099	0.168	−0.081	−0.010	−0.091	−0.148
7	0.014	0.286	0.044	−0.012	0.061	−0.043	−0.108	0.241
PC	A_2/4(1)_	A_2/5(1)_	A_2/6(1)_	A_2/7(1)_	A_2/8(1)_	A_3/4(1)_	A_3/5(1)_	A_3/6(1)_
3	−0.019	−0.075	−0.072	−0.115	0.002	−0.066	−0.141	−0.145
4	0.156	0.080	0.071	0.058	0.119	0.052	−0.068	−0.037
5	−0.186	−0.179	−0.001	−0.032	−0.038	−0.146	−0.146	0.023
6	−0.034	−0.007	−0.195	−0.156	−0.192	0.128	0.165	−0.011
7	0.050	0.036	0.026	0.001	−0.067	−0.288	−0.290	−0.296
PC	A_3/7(1)_	A_3/8(1)_	A_4/5(1)_	A_4/6(1)_	A_4/7(1)_	A_4/8(1)_	A_5/6(1)_	A_5/7(1)_
3	−0.147	−0.106	−0.114	−0.067	−0.063	−0.027	0.039	0.029
4	−0.039	0.002	−0.146	−0.133	−0.129	−0.091	0.047	0.038
5	0.007	0.007	−0.021	0.182	0.152	0.112	0.260	0.193
6	0.061	0.034	0.070	−0.117	−0.068	−0.069	−0.239	−0.159
7	−0.288	−0.278	−0.040	0.042	−0.070	−0.084	0.043	−0.057
PC	A_5/8(1)_	A_6/7(1)_	A_6/8(1)_	A_7/8(1)_	A_1/2(2)_	A_1/3(2)_	A_1/4(2)_	A_1/5(2)_
3	0.118	−0.005	0.076	0.090	−0.195	0.173	−0.041	−0.194
4	0.168	−0.044	0.006	0.096	0.041	0.010	0.285	0.191
5	0.177	−0.028	0.005	0.017	−0.008	−0.054	0.006	0.218
6	−0.185	0.117	0.049	−0.013	0.085	−0.025	−0.039	0.152
7	−0.090	−0.085	−0.124	−0.050	0.025	−0.008	−0.049	0.052
PC	A_1/6(2)_	A_1/7(2)_	A_1/8(2)_	A_2/3(2)_	A_2/4(2)_	A_2/5(2)_	A_2/6(2)_	A_2/7(2)_
3	0.057	−0.072	0.106	0.176	0.097	−0.080	0.126	0.090
4	0.068	0.117	0.125	0.061	0.235	0.211	0.046	0.138
5	0.127	0.193	0.072	0.040	−0.015	0.237	0.150	0.141
6	−0.074	0.154	−0.092	0.117	−0.108	0.141	−0.042	0.098
7	0.006	−0.089	−0.195	0.068	−0.072	0.065	−0.016	−0.003
PC	A_2/8(2)_	A_3/4(2)_	A_3/5(2)_	A_3/6(2)_	A_3/7(2)_	A_3/8(2)_	A_4/5(2)_	A_4/6(2)_
3	0.178	−0.170	−0.243	0.007	−0.158	0.006	−0.156	0.075
4	0.147	0.280	0.139	0.066	0.081	0.110	−0.051	−0.050
5	0.103	0.012	0.192	0.131	0.155	0.083	0.186	0.138
6	0.013	0.015	0.168	−0.101	0.141	−0.110	0.182	−0.037
7	0.009	−0.113	0.055	−0.027	−0.057	−0.156	0.124	0.005
PC	A_4/7(2)_	A_4/8(2)_	A_5/6(2)_	A_5/7(2)_	A_5/8(2)_	A_6/7(2)_	A_6/8(2)_	A_7/8(2)_
3	−0.014	0.135	0.158	0.194	0.171	−0.046	0.067	0.152
4	−0.163	0.009	−0.045	−0.068	0.041	−0.009	0.064	0.143
5	0.114	0.096	0.010	−0.084	−0.047	−0.058	−0.041	−0.091
6	0.115	−0.040	−0.076	0.042	−0.122	0.078	−0.027	−0.035
7	0.001	−0.172	−0.110	−0.152	−0.310	0.045	−0.178	−0.031

_(1)_ refers to the first set of sensors; _(2)_ refers to the second set of sensors.

**Table 7:** Results of prediction of discriminatory functions and class for the test sample.

Sample	Healthy	Sick	Class
41	−3.78309	−4.73564	Healthy
42	−2.84562	−5.31232	Healthy
43	−5.18519	−7.68487	Healthy
44	−2.90384	−4.85101	Healthy
45	−2.07818	−6.70706	Healthy
46	−5.69716	−4.79414	Sick
47	−9.11309	−5.06497	Sick
48	−13.2564	−4.44323	Sick
49	−13.2564	−4.44323	Sick
50	−6.79918	−2.24365	Sick

## Supplementary Materials

The following are available online at https://www.mdpi.com/article/10.3390/vetsci8050074/s1: Figure S1. PCA–LDA plot.emf; Table S1. Autoscaled sensor data.xlsx.
